# Late-Night Feeding, Sleep Disturbance, and Nocturnal Congestion Mediated by Hyperglycemia, Renal Sodium Retention, and Cortisol: A Narrative Review

**DOI:** 10.3390/clockssleep8010001

**Published:** 2025-12-24

**Authors:** Ronald B. Brown

**Affiliations:** Waterloo Institute for Complexity and Innovation, University of Waterloo, Waterloo, ON N2L 3G1, Canada; r26brown@uwaterloo.ca

**Keywords:** time-restricted feeding, circadian metabolism, renal sodium retention, plasma osmolarity, hypothalamic–pituitary–adrenal axis, nasal congestion, sleep fragmentation, obstructive sleep apnea, sleep-disordered breathing, nocturia

## Abstract

Late-night feeding, defined in the present review as feeding after 8:00 pm when evening insulin secretion and sensitivity are low, is increasingly prevalent in Western society and is recognized as a disruptor of metabolic homeostasis. Yet health problems related to late-night feeding are largely ignored in time-restricted feeding studies that generally do not extend past an 8:00 pm feeding window. This paper proposes a novel cascade linking late-night hyperglycemia with sleep disturbances and nasal congestion mediated by renal sodium retention, increased plasma osmolarity, and stress hormone release by hypothalamic–pituitary–adrenal axis activation. The narrative describes the circadian decline in insulin sensitivity, which amplifies postprandial glucose surges following late-night feeding. Elevated glucose levels drive renal glucose reabsorption via sodium–glucose cotransporters, promoting sodium retention independent of insulin. Increased sodium retention raises extracellular osmolarity, activating hypothalamic osmoreceptors and stimulating the hypothalamic–pituitary–adrenal axis. Cortisol release promotes alertness, while fluid retention and mucosal edema contribute to nasal congestion and early waking. Supine fluid redistribution during sleep further exacerbates airway narrowing, increasing the risk of sleep fragmentation and obstructive sleep apnea. The present paper fills a gap in current time-restricted feeding literature by integrating renal, osmotic, and neuroendocrine pathways that may be overlooked as underlying mechanisms of dysregulated glucose control and hormone dysfunction. Reviewed evidence suggests that symptoms such as nocturnal congestion and sleep disruption are not merely incidental to late-night feeding but frame late night feeding as a risk factor with underlying physiological stressors that could contribute to cardiometabolic risk.

## 1. Introduction

An internal circadian clock in most organisms regulates daily cycles of sleep and wakefulness, feeding and fasting, and metabolism and hormone release [1]. Misalignment of feeding with circadian cycles reduces glucose tolerance (i.e., raises postprandial glucose levels) [2], and in combination with misaligned sleeping, has been shown to have adverse metabolic and cardiovascular effects [3]. Time-restricted feeding (TRF) aims to align dietary intake with circadian cycles by consolidating food intake within a daily window, typically 8 to 10 h [4]. A 2024 review, however, suggests that TRF studies generally do not extend feeding windows past 8:00 pm [5]. Consequently, the present review defines “late-night feeding” as food intake occurring after 8:00 pm. This later feeding time is considered misaligned with the body’s circadian rhythm, which favors earlier feeding windows. For example, a dietary intervention using early TRF improved cardiometabolic health with benefits in glycemic control and insulin sensitivity independent of food intake and weight loss [6].

Recently, shifts in work schedules, digital engagement, and social norms have contributed to a growing trend of delayed eating, with more Americans consuming meals or snacks well into the evening hours [7]. Additionally, night eating syndrome (NES) is an eating disorder associated with depression in which patients with obesity were found to consume 25% or more of their daily caloric intake after dinner [8]. While TRF studies emphasize the benefits of early feeding, TRF studies rarely appear to isolate the metabolic consequences and insulin dynamics during late-night feeding. Furthermore, TRF studies with endpoints such as fasting glucose, insulin resistance, and body weight, do not fully investigate the underlying causative mechanisms of observed effects. In particular, the adverse physiological consequences of late-night feeding and circadian misalignment—including subjective symptoms like nasal congestion, early waking, and sleep fragmentation—remain largely unexplored. These symptoms may be overlooked as incidental or unrelated to metabolic function, yet they likely reflect deeper disruptions in renal, osmotic, and neuroendocrine physiology.

**Figure 1:**
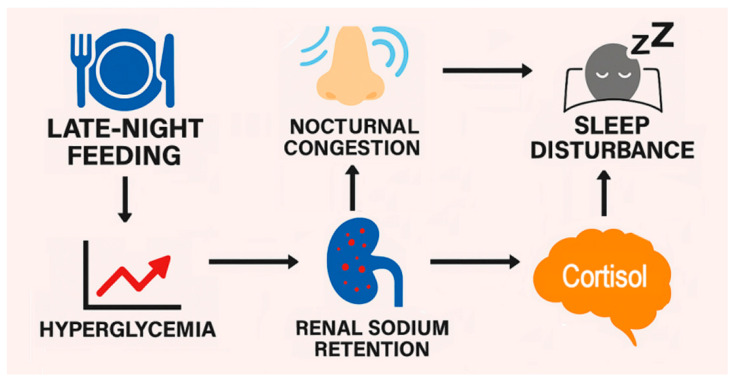
The association of late-night feeding with sleep disturbance is mediated by hyperglycemia and renal sodium retention, which contributes to cortisol release and nocturnal nasal congestion.

This narrative review proposes a novel mechanistic cascade linking late-night feeding to sleep disturbance and mucosal congestion mediated by hyperglycemia, renal sodium retention, and increased plasma osmolarity that triggers stress hormone release (including cortisol) via activation of the hypothalamic–pituitary–adrenal (HPA) axis. This cascade is illustrated in Figure 1. The paper posits that this pathway not only explains common subjective complaints following late-night meals but also reveals hidden stressors that frame late night feeding as a risk factor affecting metabolic and cardiovascular health and quality of life. The foundational cause of late-night feeding effects lies in the circadian decline in insulin sensitivity. Research shows that as the day progresses, the body’s ability to respond to insulin diminishes, leading to larger postprandial glucose surges in the evening versus midday [9]. This elevation in glucose triggers renal glucose reabsorption via sodium–glucose cotransporter-2 (SGLT2), a process that is tightly coupled with sodium retention [10]. Unlike insulin-mediated renal sodium reabsorption [11], which predominates during daytime feeding, renal glucose reabsorption via SGLT2 operates independently of insulin [12] and could become more prominent when insulin levels are low—for example, during late-night meals.

Sodium retention increases extracellular fluid volume and raises plasma osmolarity, which activates osmoreceptors in the hypothalamus [13]. This stimulation leads to the release of corticotropin-releasing hormone (CRH) and arginine vasopressin (AVP), initiating a neuroendocrine cascade that culminates in cortisol release from the adrenal cortex. Cortisol promotes alertness and impairs sleep architecture [14] which is associated with combined symptoms of daytime fatigue and hyperarousal [15], referred to in a public online forum as “tired but wired” [16]. Sodium retention in the context of sleep also has profound implications. For example, supine posture during sleep facilitates fluid redistribution toward the upper body, promoting mucosal edema and narrowing of the nasal passages, leading to increased airway resistance, mouth breathing, and a higher risk of obstructive sleep apnea (OSA) [17]. The overall result of late-night feeding is a constellation of symptoms: nasal congestion, early waking, and an alert sensation that undermines sleep quality.

By integrating renal, osmotic, and neuroendocrine pathways, the narrative review in this paper expands the scope of TRF physiology beyond glucose metrics. The paper uses a grounded theory literature review method to synthesize evidence from findings of peer-reviewed research [18]. Starting with a clean slate to remove assumptions and increase objectivity, the method uses an iterative and rigorous process to select research findings for inductive synthesis (generating principles from data) of thematic concepts, associations, and causative mechanisms underlying subjective symptoms in late-night feedings. Selected studies were searched using Google, Google Scholar, PubMed, and Web of Science, based on combinations of keywords listed at the beginning of this paper. However, unlike the rigorous rules of a systematic review, the search technique in a grounded theory literature review is much more flexible and open-ended, prompting the researcher to freely search along the trail of evidence as it forms iteratively. In qualitative research, the researcher often relies on personal judgment, experience, and knowledge of the research topic to guide the collection and synthesis of data. Results of the synthesis in the present paper aim to enable researchers to capture the full spectrum of metabolic and experiential outcomes of late-night eating and address late-night feeding consequences with future TRF interventions.

## 2. The Internal Circadian Clock

The human body’s built-in 24-h rhythm known as the circadian clock is anchored in the suprachiasmatic nucleus (SCN) of the hypothalamus, a small region just above the optic chiasm that receives light signals from the retina to synchronize internal timekeeping with the external day–night cycle [19]. At the cellular level, the circadian clock is driven by a self-regulating 24-h feedback loop of clock genes. The proteins circadian locomotor output cycles kaput (CLOCK) and brain and muscle arnt-like protein-1 (BMAL1) activate gene transcription of *Period* (*Per1–3*) and *Cryptochrome* (*Cry1–2*), whose protein products accumulate in the nucleus and suppress their own gene transcription [20]. Additional regulation by genes such as *REV-ERBα* and *RORα* fine-tune this loop and link it to metabolic pathways, including glucose and lipid handling [21].

The SCN sends timing signals to entrain or synchronize peripheral clocks in organs like the liver, kidney, and adipose tissue, which maintain their own rhythmic gene expression and physiological outputs [22]. These peripheral clocks are sensitive not only to light but also to feeding times, sleep patterns, and metabolic cues. For example, irregular feeding times can uncouple peripheral clocks from the SCN, leading to misalignment between central and local rhythms. This misalignment has clinical consequences. Circadian disruption—caused by shift work, late-night eating, or fragmented sleep—impairs glucose metabolism, alters renal sodium handling, and increases cardiometabolic risk [23]. Renal glucose reabsorption by SGLT2, which operates independently of insulin, is especially amplified during the night when lower levels of insulin decrease insulin-mediated sodium reabsorption in the nephron [24]. The result is fluid retention, elevated blood pressure, and disrupted sleep architecture—especially when compounded by neuroendocrine activation and postural fluid shifts.

## 3. Time-Restricted Feeding and Circadian Alignment

TRF aligns eating patterns with the body’s internal circadian clock, which governs metabolic processes such as glucose regulation, hormone secretion, and energy expenditure [25]. At the molecular level, TRF influences the expression of clock genes in peripheral tissues, including the liver, pancreas, and adipose tissue where food intake acts as a zeitgeber or external cue that entrains peripheral clocks [26]. Genes in peripheral clocks regulate insulin sensitivity, lipid metabolism, and inflammatory pathways.

Meals timed to occur earlier in the day—a pattern known as early TRF (e-TRF) with the first meal before 10:30 am -- reinforce the natural rhythm of metabolic activity, which peaks in the morning and declines toward evening [27]. Indeed, a 2018 review of glucose regulation by the circadian system confirms that glycemic control in healthy adults is poorer in the evening and at night, driven by diurnal rhythms of insulin secretion and peripheral insulin sensitivity which peak in the morning [9]. Furthermore, late TRF (l-TRF) with the first meal after 11:30 am may conflict with circadian signals when compared to e-TRF, with benefits in glucose resistance reported more often for e-TRF [27]. Importantly, the negative metabolic effects of l-TRF could have implications on adverse metabolic effects of daily fasting until noon, or the extended postabsorptive state, which is associated with asynchrony of circadian clock gene expression and dysregulated glucose metabolism [28]. A 2022 clinical trial confirms that daily fasting until noon, about 5 h after wake time, is associated with later feeding into the evening [29].

Turning to late-night feeding studies, a 2024 analysis of data from the National Health and Nutrition Examination Survey (2002–2018) found increased risks of all-cause mortality, cancer mortality, and diabetes mortality associated with night-time eating after 10 pm, which the researchers speculated was due to circadian disruption that contributed to mortality risk [30]. A 2020 clinical trial found that nocturnal glucose intolerance, higher evening cortisol, and reductions in the oxidation and mobilization of fatty acids in healthy participants was induced by a late dinner after 10 pm [31]. Increased cortisol, systemic inflammation, sleep disturbance, and suppressed serotonin and melatonin production that exacerbated mood disorders like depression were also found in a 2025 review examining emotional health impacts from circadian disruption by late-night eating, which the researchers defined as eating after 9:00 pm [32].

Summing up, clinical studies have shown that TRF can improve markers of metabolic health, including reductions in body weight, blood pressure, and insulin resistance—even without calorie restriction. These benefits appear to be more pronounced with e-TRF, which supports the body’s natural hormonal rhythms and reduces overnight glucose excursions. Importantly, TRF also lowers the demand for late-night insulin, which may reduce renal sodium retention and fluid overload associated with glucose excursions— mechanisms relevant to sleep quality and cardiometabolic risk. From a practical standpoint, TRF offers a simple, behaviorally sustainable framework for improving health. By avoiding food intake during the biological night—when melatonin levels are high and insulin sensitivity is low—TRF helps prevent metabolic disruption and supports restorative sleep. Individuals who adopt TRF have reported improved energy [4], appetite control [33], and better adherence to healthy eating patterns [34]. However, the effectiveness of TRF may depend on individual chronotype (preference for early or late hours), sleep timing, as well as age and metabolic status. Future studies should explore personalized TRF schedules that account for these variables, especially in populations at risk for obesity, diabetes, and sleep disorders.

## 4. Mechanistic Framework

The following subsections describe in greater detail the mechanisms underlying adverse metabolic effects and symptoms associated with late-night feeding.

### 4.1. Circadian Decline in Insulin Sensitivity and Postprandial Glucose Excursions

Insulin sensitivity follows a robust circadian rhythm, peaking in the morning and declining throughout the day due to synchronized oscillations in peripheral clock genes and hormonal regulators [2,35]. This decline is not merely behavioral but reflects intrinsic changes in glucose transporter expression, insulin receptor signaling, and hepatic glucose uptake [3]. Studies using gold standard methods for glucose monitoring, such as the oral glucose tolerance test (OGTT), confirm that evening meals produce significantly higher postprandial glucose excursions than identical meals consumed earlier in the day—even in healthy individuals [6,36].

Glucokinase (GCK), a key glucose sensor in hepatocytes and pancreatic β-cells, is regulated by CLOCK and BMAL1 transcription factors, classifying it as a clock-controlled gene [37]. Its activity declines in the evening, impairing hepatic glucose clearance from the blood and insulin secretion. Moreover, incretin hormones secreted in the gut to increase insulin sensitivity, such as glucagon-like peptide-1 (GLP-1) and glucose-dependent insulinotropic polypeptide (GIP), exhibit diurnal variation with reduced secretion and receptor sensitivity in the evening, further blunting insulin response [38]. This circadian misalignment amplifies hyperglycemia following late-night meals, setting the stage for renal and neuroendocrine consequences. Importantly, this elevation in glucose occurs even in the absence of overt insulin resistance, highlighting the need for TRF protocols that align feeding with circadian metabolic capacity.

### 4.2. Renal Glucose–Sodium Co-Reabsorption via SGLT2 and Sodium Retention

In the proximal tubule, glucose and sodium are co-transported via SGLT2, which reabsorbs ≥ 80% of filtered glucose under normal conditions [39]. This process is tightly coupled: for every glucose molecule reabsorbed, a sodium ion is retained. During late-night hyperglycemia, filtered glucose load increases, enhancing SGLT2-mediated reabsorption and promoting sodium retention—even when insulin levels are low.

Unlike insulin-driven sodium reabsorption in the renal distal tubule via the epithelial sodium channel (ENaC) and sodium-potassium adenosine triphosphatase (Na^+^/K^+^-ATPase) [40], which predominates during daytime feeding, SGLT2-mediated sodium retention is insulin-independent and becomes dominant when insulin secretion is blunted [41]. This mechanism is also relevant in insulin-resistant states as increases in SGLT2 activity and gene expression are likely due to elevated plasma glucose concentrations [42] with parallel increases in related sodium retention.

Delaying early evening feeding until the late night can also contribute to nocturnal glucose excursions through postprandial hyperglycemia after meal skipping, in which the rise in glucose levels is exaggerated in a second meal after a missed meal [43]. Although often attributed to an impaired insulin response, this phenomenon may involve a prolonged-fasting mechanism that maintains serum glucose through gluconeogenesis from catabolism of glycerol, lactate, and glucogenic amino acids, and from hepatic glycogenolysis activated by glucagon [44,45]. Consuming a second meal before gluconeogenesis subsides can overload the system, producing higher postprandial glucose from both endogenous and exogenous sources. Consequently, increased SGLT2 activation and sodium retention are likely aligned with higher glucose levels when an earlier evening meal is delayed until the late night. Recent studies show that SGLT2 inhibitors not only improve glycemic control but also reduce extracellular fluid volume and blood pressure [46], underscoring the physiological significance of glucose-induced sodium retention. Moreover, sodium/hydrogen exchanger-3 (NHE3), another sodium transporter in the proximal tubule, is upregulated by hyperglycemia and contributes to sodium retention and fluid overload [47]—particularly likely in the context of late-night feeding. This renal sodium retention increases plasma osmolarity and extracellular fluid volume, triggering osmoregulatory and neuroendocrine responses that impair sleep and respiratory stability. Future research should investigate the use of SGLT2 inhibitors to treat sleep disturbances with nasal congestion.

### 4.3. Plasma Osmolarity, HPA Axis, Cortisol, and Nocturia

Sodium retention elevates plasma osmolarity, which is sensed by osmoreceptors in the organum vasculosum of the lamina terminalis (OVLT) and the subfornical organ—circumventricular structures that lack a blood–brain barrier [48]. These receptors stimulate the hypothalamus to release CRH and AVP, initiating the HPA axis cascade.

CRH and AVP act synergistically on the anterior pituitary to release adrenocorticotropic hormone (ACTH), which stimulates cortisol secretion from the adrenal cortex [49]. Cortisol promotes alertness, impairs sleep architecture, and enhances sodium retention via mineralocorticoid receptor activation—creating a feedback loop that amplifies osmotic and neuroendocrine stress.

Elevated cortisol levels are associated with reduced slow-wave sleep, increased sleep fragmentation, and early morning arousals [50]. These effects are particularly pronounced when cortisol rises during biological sleep, as occurs following late-night feeding [51]. Moreover, AVP, also known as antidiuretic hormone (ADH), contributes to fluid retention and mucosal edema [52], compounding the impact on nasal airflow and sleep quality.

Nocturia, the need to void urine during the night, is also associated with poor sleep quality [53], and suggestions to manage nocturia include optimal “timing, amount, and type of fluid ingested” [54]. Nocturia can also sometimes cause difficulties in returning to sleep [55], inferring that related factors may keep an individual awake at night. Indeed, HPA-axis activation elevates nighttime cortisol levels and is associated with restless and fragmented sleep [56], and eating at night raised overnight cortisol levels in nightshift workers [51]. These findings suggest that late-night feeding may increase sleep disturbance associated with nocturia through HPA-axis activation and cortisol-mediated wakefulness. Nocturia is also associated with frequent daytime napping [57], and frequent daytime napping is associated with nighttime sleep disturbance [58]. Taken together, the cascade of late-night feeding leading to sleep disturbance and napping could contribute to the cultural tradition of siestas and daytime napping in countries that consume late-evening dinners [59].

### 4.4. Nocturnal Rostral Fluid Shift and Sleep-Disordered Breathing

During sleep, especially in the supine position, fluid redistributes from the lower extremities to the head and neck, causing a nocturnal rostral fluid shift [60]. This fluid shift exacerbates mucosal edema in the nasal passages, which may be already primed by inflammatory-induced vasodilation, edema, and swelling [61]. The result is nasal congestion, reduced airflow, and increased upper airway resistance which contribute to sleep-disordered breathing (SDB) [62], a major determinant of morbidity and mortality, having a high rate of prevalence [63].

Nasal obstruction promotes mouth breathing, destabilizing the airway and increasing the risk of OSA. Chronic sinusitis and mucosal inflammation further narrow the airway, compounding the effects of congestion [64,65]. Sleep fragmentation from congestion and apnea activates the HPA axis [66], reinforcing the cycle of neuroendocrine stress and metabolic disruption. Relatedly, daily sodium intake in patients with heart failure was correlated with higher apnea–hypopnea index (AHI) scores (the number of times breathing is interrupted per hour of sleep), attributed to sodium-induced fluid retention and nocturnal rostral shift of fluid to the neck area which narrowed the upper airway [67]. These findings support the hypothesis that late-night feeding could exacerbate airway instability via fluid dynamics and neuroendocrine activation.

Future studies should investigate specific dietary factors in late-night feeding associated with SDB—for example, capsaicin from chili peppers has been shown to cause neurogenic inflammation leading to nasal tissue edema, swelling, plasma leakage, and leukocyte infiltration which subsequently causes nasal congestion [68]. Furthermore, morning nasal congestion is highly prevalent within the population, and symptoms of nasal congestion in allergic rhinitis (AR) are often worse in the morning [69], Studies should examine circadian-related mechanisms linking late-night feeding with morning nasal congestion.

### 4.5. Sodium Retention, Sinus Congestion, and Mucosal Edema

Late-night feeding, particularly after 8:00 pm may trigger systemic fluid dysregulation that manifests as nasal mucosal edema and sinus congestion. For example, meals consumed during the biological night are more likely to elevate postprandial glucose, which activates renal sodium retention via aldosterone, vasopressin, and sympathetic pathways [70]. Sodium retention expands extracellular fluid volume and increases hydrostatic pressure, which can affect low-resistance vascular beds such as the nasal submucosa [71]. Simultaneously, hyperglycemia raises plasma osmolality and impairs endothelial integrity [72], potentially promoting capillary leak and fluid extravasation into mucosal tissues. The nasal mucosa, with its dense venous plexus and loose connective architecture, is particularly sensitive to these shifts, resulting in rapid swelling and congestion [61]. Inflammatory mediators further amplify this response: both sodium and glucose excess stimulate cytokine release, e.g., tumor necrosis factor-α (TNF-α), interleukin-6 (IL-6)—which increases vascular permeability and neurogenic vasodilation (blood vessel widening caused by nerve signals) [61]. These mechanisms suggest that sinus congestion may reflect a systemic consequence of late-night feeding, linking dietary timing to mucosal symptoms via renal and glycemic pathways.

### 4.6. Summary Cascade

**Figure 2:**
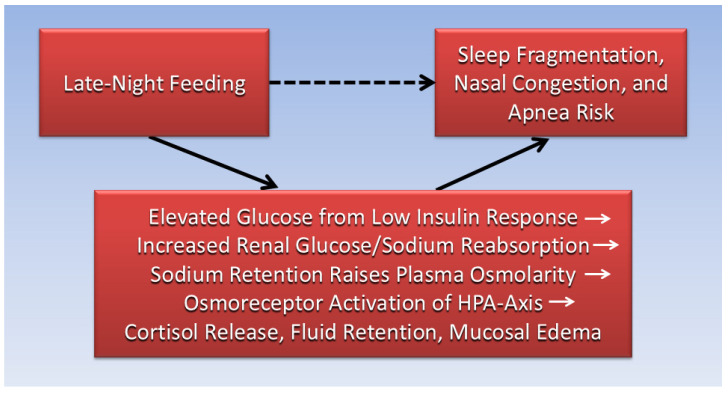
The association (dotted arrow) of late-night feeding with sleep fragmentation, nasal congestion, and apnea risk is mediated within the causal pathway (solid arrows) by elevated glucose from low insulin response, increased renal glucose/sodium reabsorption, sodium retention that raises plasma osmolarity and activates the HPA-axis, causing cortisol release, fluid retention, and mucosal edema.

Figure 2 illustrates mediators in a causative pathway that integrate metabolic, renal, and neuroendocrine physiology to explain how late-night feeding is associated with disrupted sleep and nocturnal congestion. This knowledge expands TRF literature by revealing mechanisms that are often overlooked yet clinically relevant.

## 5. Implications for Time-Restricted Feeding Protocols

The mechanistic cascade outlined in this narrative review has direct implications for the design and optimization of time-restricted feeding (TRF) protocols. The following recommendations apply to future TRF protocols:

1. Prioritize earlier feeding windows (e.g., 08:00 am–4:00 pm or 10:00 am–6:00 pm) to align with peak insulin sensitivity and minimize renal sodium retention [5,6,27].
2. Monitor subjective symptoms such as nasal congestion, sleep quality, and early waking as indicators of osmotic and neuroendocrine stress [14,15,61].
3. Incorporate mechanistic biomarkers (e.g., plasma osmolarity, cortisol, ACTH, AVP) to validate symptom–physiology linkages [13,49,50,52].
4. Tailor protocols to individual physiology, accounting for anatomical risk factors (e.g., airway structure, neck circumference) and metabolic status (e.g., insulin resistance, fluid retention) [24,62,63,65].
5. Avoid high-sodium or high-glycemic meals late in the feeding window, especially in individuals with sleep vulnerability or fluid-sensitive conditions [30,31,67,70].

In addition, TRF studies should adopt mixed-methods approaches that combine quantitative biomarkers with qualitative symptom tracking. Grounded theory analysis of participant narratives can reveal patterns that are missed by standard questionnaires, offering deeper insight into the lived experience of TRF adherence. In summary, optimizing TRF protocols requires a shift from outcome-based design to mechanistic precision. By addressing the renal and neuroendocrine consequences of late-night feeding, researchers can enhance protocol efficacy and adherence—advancing TRF from a behavioral intervention to a systems-level metabolic therapy. Studies are needed to assess the clinical application and effect of these TRF protocols on quality of life, reduced metabolic disturbances, and lowered cardiometabolic risk in patients of all ages with related conditions such as diabetes, metabolic syndrome, obesity, etc.

The following list includes examples of unsolved questions that require further investigations in the field of late-night feeding, circadian metabolism, and metabolic health:

1. What are the predisposing lifestyle and behavioral health factors that increase an individual’s susceptibility to symptoms and metabolic effects from late-night feeding?
2. What influence does physical activity have on effects of late-night feeding?
3. How do different dietary patterns and specific foods interact with late-night feeding to cause sleep and metabolic disturbances?
4. How does late-night feeding interact with mental stress and behavioral disorders?
5. Do changes in circadian metabolism related to seasonality in temperate zones alter effects of late-night feeding?
6. How should dietary guidelines incorporate TRF to reduce population level metabolic risk associated with late-night feeding?

## 6. Summary and Conclusions

The present paper proposes a novel mechanistic cascade that frames late-night feeding as a risk factor for sleep disruption and nasal congestion via renal sodium retention, increased plasma osmolarity, and hypothalamic–pituitary–adrenal (HPA) axis activation. While TRFs are often framed as a behavioral intervention to improve glycemic control, the present review proposes reframing TRF as a systems-level metabolic therapy—one that must account for renal, neuroendocrine, and respiratory physiology to optimize outcomes.

Importantly, this framework challenges the assumption that the size of the feeding window alone determines TRF efficacy. While early e-TRF protocols consistently outperform l- TRF in terms of glycemic control and insulin sensitivity, the proposed framework suggests that these benefits may also stem from reduced activation of renal and neuroendocrine stress pathways. This insight has practical implications: shifting the feeding window earlier in the day may improve sleep quality and respiratory stability, even if total caloric intake remains unchanged.

The proposed framework also highlights the need for individualized TRF protocols. Anatomical risk factors (e.g., narrow nasal passages, high neck circumference), metabolic status (e.g., insulin resistance, fluid retention), and subjective symptom profiles should inform feeding window design. For example, individuals prone to nasal congestion or sleep apnea may benefit from e-TRF schedules that minimize late-night sodium retention and mucosal edema.

Future research should validate the proposed framework using mechanistic biomarkers such as plasma osmolarity, cortisol, ACTH, AVP, and neck circumference. Sleep studies incorporating polysomnography, nasal airflow metrics, and symptom diaries can further elucidate the link between feeding timing and respiratory stability.

In conclusion, this narrative review expands the scope of TRF physiology by integrating renal, osmotic, and neuroendocrine pathways that are rarely considered in metabolic studies. It reframes subjective symptoms as mechanistically informative signals of physiological stress and proposes a systems-level model to optimize TRF protocols. By moving beyond glucose metrics to include sleep quality, airway stability, and neuroendocrine activation, TRF can be transformed from a behavioral intervention into a precision metabolic therapy. Ultimately, optimizing TRF requires more than adjusting the clock—it demands a deeper understanding of how feeding timing interacts with renal physiology, neuroendocrine stress, and airway dynamics. This review lays the groundwork for a more comprehensive, mechanistically grounded approach to advance chrononutrition, the study of how timed feeding interacts with circadian rhythms that affect metabolic health.

## Data Availability

No new data were created or analyzed in this study.

## Abbreviations

The following abbreviations are used in this manuscript:

TRF	Time-restricted feeding
e-TRF	Early time-restricted feeding
l-TRF	Late time-restricted feeding
NES	Night eating syndrome
HPA	Hypothalamic–pituitary–adrenal
SGLT2	Sodium-glucose cotransporter-2
AVP	Arginine vasopressin
CRH	Corticotropin-releasing hormone
OSA	Obstructive sleep apnea
SCN	Suprachiasmatic nucleus
SDB	Sleep-disordered breathing
CLOCK	Circadian locomotor output cycle kaput
BMAL1	Brain and muscle arnt-like protein-1
OGTT	Oral glucose tolerance test
GCK	Glucokinase
GLP-1	Glucagon-like peptide-1
GIP	Glucose-dependent insulinotropic polypeptide
Na^+^/K^+^-ATPase	Sodium-potassium adenosine triphosphatase
ENaC	Epithelial sodium channel
NHE3	Sodium/hydrogen exchanger-3
OVLT	Organum vasculosum of the lamina terminalis
ACTH	Adrenocorticotropic hormone
AHI	Apnea–hypopnea index
TNF-α	Tumor necrosis factor-α
IL-6	Interleukin-6
AR	Allergic rhinitis
